# High-Glucose Media Reduced the Viability and Induced Differential Pro-Inflammatory Cytokines in Human Periodontal Ligament Fibroblasts

**DOI:** 10.3390/biom13040690

**Published:** 2023-04-19

**Authors:** Alaa Aldoss, Rhodanne Lambarte, Fahd Alsalleeh

**Affiliations:** 1 Restorative Dental Sciences, College of Dentistry, King Saud University, P.O. Box 60169, Riyadh 11545, Saudi Arabia; 2 Dental University Hospital, King Saud University, P.O. Box 60169, Riyadh 11545, Saudi Arabia; 3 Molecular and Cell Biology Laboratory, Prince Naif Bin AbdulAziz Health Research Center, College of Dentistry, King Saud University Medical City, P.O. Box 60169, Riyadh 11545, Saudi Arabia

**Keywords:** diabetes mellitus, glucose, hyperglycemia, inflammatory response, periodontitis, periodontal ligament fibroblast

## Abstract

Hyperglycemic condition in diabetic patients tends to exacerbate periodontitis severity. Thus, the influence of hyperglycemia on the biological and inflammatory response of periodontal ligament fibroblasts (PDLFs) needs to be elucidated. In this study, PDLFs were seeded in media containing glucose concentrations (5.5, 25, or 50 mM) and stimulated with 1 µg/mL of lipopolysaccharide (LPS). PDLFs’ viability, cytotoxicity, and the migration ability were determined. The mRNA expression of Interleukin (IL)-6, IL-10, and IL-23 (p19/p40), and Toll-like receptor (TLR)-4 were analyzed; at 6 and 24 h, protein expression of IL-6 and IL-10 was also determined. PDLFs grown in 50 mM glucose medium showed lower viability. The 5.5 mM glucose led to the highest percentage of wound closure compared to 25 mM and 50 mM glucose with/without LPS. Additionally, 50 mM glucose with LPS exhibited the least migration ability among all groups. The expression of IL-6 was amplified significantly in LPS-stimulated cells in 50 mM glucose medium. IL-10 was constitutively expressed in different glucose concentrations, and LPS stimulation decreased it. IL-23 p40 was up-regulated after LPS stimulation in 50 mM glucose concentration. TLR-4 was highly expressed after LPS stimulation in all glucose concentrations. Hyperglycemic conditions limit PDLF proliferation and migration, and enhance the expression of certain pro-inflammatory cytokines to induce periodontitis.

## 1. Introduction

Diabetes mellitus (DM) is “a group of metabolic diseases characterized by hyperglycemia resulting from defects in insulin secretion, resistance to insulin action, or both” [1]. Persistent hyperglycemia tends to up-regulate the inflammatory mediators leading to damage and failure of different tissues and organs [2]. In addition, chronic elevation of blood glucose levels leads to some significant changes in oral tissues, including xerostomia, opportunistic fungal infections, poor healing of oral mucosa, increased incidence and severity of caries, and increased prevalence of periodontal diseases and apical periodontitis [3,4].

Periodontitis is a chronic inflammatory disease due to the interaction between microbial agents and the host immune response, resulting in the inflammation and destruction of the tooth-supporting structure [5]. The interrelation between DM and periodontitis is well recognized. Clinical studies have shown a significant increase in the prevalence and severity of periodontal disease among individuals with diabetes compared to non-diabetic ones [6,7,8]. At the same time, animal studies have revealed that periodontitis significantly enhances the development of insulin resistance and pancreatic β-cell dysfunction in DM animal models [9,10]. The degree of glycemic control is a crucial determinant in this relationship. It shows that persistent hyperglycemia may induce an inflammatory response, leading to a significant progression of gingivitis and periodontitis in diabetic patients, and the presence of one condition tends to increase the incidence and intensity of the other [11]. Surprisingly, periodontal therapy in diabetic patients tends to positively affect both diseases [6,12,13,14].

Periodontal ligament fibroblasts (PDLFs) play a crucial role in forming, maintaining, and remodeling the periodontal fibrous tissue connecting teeth to the alveolar bone [15]. PDLFs have an active role in innate immune responses to invading microorganisms. They can effectively initiate an inflammatory response by activating pattern-recognition receptors, such as Toll-like receptors (TLRs), that bind to pathogens or their virulence factors such as lipopolysaccharide (LPS). Subsequently, the expression of pro-inflammatory cytokines followed by a wide range of immune events will induce inflammation [16,17,18].

The hyperglycemic conditions in diabetic patients presumably influence the biological and inflammatory response of PDLFs. Recent studies have used a wide range of glucose media (5.5–50 mM) to study their effects on PDLFs. A glucose concentration of 5.5 mM simulates the normal blood glucose level in vivo, whereas glucose concentrations ranging from 25 to 50 mM have been mostly used in previous studies in cell cultures to mimic high blood glucose levels in diabetic conditions, evaluating different outcomes [19,20,21]. In fact, a severely high-glucose concentration (45 mM) inhibited the cellular proliferation and osteogenic differentiation of PDLFs [22,23]. However, a high-glucose concentration (25 mM) did not significantly affect the proliferation but induced further osteogenic differentiation compared to the normal glucose concentration (5.5 mM) [24].

Bacterial lipopolysaccharides (LPS) are the main virulence factor of Gram-negative bacteria involved in periodontitis. It is an essential inflammatory stimulus that can impair the junctional epithelial barrier and basement membrane, initiating inflammation and leading to periodontal tissue destruction [25]. Previous in vitro studies have challenged periodontal cells with LPS at various concentrations from different bacterial sources, such as Porphyromonas gingivalis and *Escherichia coli* (*E. coli*) [18,26]. Both sources of LPS have demonstrated a differential inflammatory response, but *E. coli* LPS induces a stronger cytokine and chemokine response in gingival fibroblasts [26,27,28]. LPS alone has been shown to significantly reduce the viability of PDLFs and induce the expression of different pro-inflammatory mediators [29]. At the same time, hyperglycemia presence will disrupt the host defense, which may lead to an overexpression of pro-inflammatory cytokines, mediating host-tissue damage [11,30]. One study showed IL-6 expression to be significantly up-regulated when PDL cells were treated with 45 mM glucose [20]. However, the interaction effects of different glucose concentrations on PDLFs in the presence of bacterial stimulus need to be further evaluated.

PDLFs respond differently to different glucose concentrations. Therefore, studying the interaction effects of LPS and different glucose concentrations on PDLF proliferation, cytotoxicity, migration, and inflammatory response may advance the current understanding of the pathogenic mechanism of periodontitis in diabetic patients. Accordingly, the central aim of this investigation was to determine the effect of different glucose concentrations on the proliferation, cytotoxicity, wound healing ability of human PDLFs, and the expression of selected inflammatory cytokines, Interleukin (IL)-6, IL-10, and IL-23 (p19/p40), and Toll-like receptor (TLR)-4, with and without stimulation with LPS.

## 2. Materials and Methods

### 2.1. Isolation and Culturing of Human Periodontal Ligament Fibroblasts (PDLFs)

Human primary PDLFs were isolated from the healthy periodontal tissue of caries-free premolars planned for extraction for orthodontic reasons. Six healthy patients (two males and four females) between 18–25 years of age voluntarily signed the written informed consent before the extraction, according to the protocol approved by King Saud University Institutional Review Board (Research Project No. E-20-4963).

PDLFs were isolated based on a previously published method [18]. Briefly, PDL tissues were gently scraped from the surface of the middle third of the tooth root and then cut into small pieces, vibrated, and disaggregated with 0.1% collagenase I (Sigma-Aldrich, St. Louis, MO, USA) for 30 min at 37 °C. The PDL tissue explants were then inoculated into a 60 mm culture dish (Corning Incorporated, Corning, New York, NY, USA) and grown on a complete Dulbecco-modified Eagle’s medium (Thermo Fisher Scientific, Waltham, MA, USA). This medium was supplemented with 10% heat-inactivated fetal bovine serum (Thermo Fisher Scientific) and a 1% antibiotic/antimycotic solution containing 100 U/mL penicillin G, 100 µg/mL streptomycin, and 0.025 µg/mL amphotericin B (Thermo Fisher Scientific). The cells were maintained under standard cell culture conditions (37 °C, 100% humidity, 95% air, and 5% CO_2_), and the culture medium was replaced every 2–3 days. Migrating cells from the PDL tissue explants were observed under a microscope after 21–25 days of culture. The pool of cells from each patient was subcultured at 70–80% confluence, and the fourth–sixth passage cells were used in the following experiments.

### 2.2. Immunohistochemistry

Immunohistochemistry performed the identification of PDLFs to determine the expression of keratin and vimentin proteins. In brief, PDLFs were seeded onto 6-well plates (Thermo Fisher Scientific) at a seeding density of 1 × 10^6^ cells/well. Next, cells were gently rinsed with phosphate-buffered saline (PBS) containing 5% bovine serum albumin three times and fixed with 4% paraformaldehyde for 10 min. Next, the cells were incubated using a monoclonal cytokeratin antibody (Elabscience^®^ Biotechnology, Texas, TX, USA) as the primary antibody for 1 h, followed by treatment with goat anti-rabbit IgG (e-Lab science) as the secondary antibody for 1 h. For vimentin staining, a monoclonal antibody (e-Lab science) was used as a primary antibody for 1 h, followed by treatment with goat anti-mouse IgG (e-Lab science) as a secondary antibody for 1 h. Finally, the chromogenic reaction was performed with a Pierce™ diaminobenzidine tetrahydrochloride (DAB) substrate kit (Thermo Fisher Scientific) to visualize immunoreaction, followed by counterstaining with hematoxylin [19].

### 2.3. Stimulation of PDLFs with/without Lipopolysaccharide in Different Glucose Concentrations

PDLFs that reached confluence in the culture medium were collected, washed, and counted with a hemocytometer. Cells were subjected to three complete culture mediums with three glucose concentrations (D-glucose, Sigma-Aldrich): normal glucose at 5.5 mM, high glucose at 25 mM, and severe high glucose at 50 mM with or without 1 µg/mL of *E. coli* LPS (Sigma-Aldrich) stimulation [19,21,24,31].

### 2.4. Alamar Blue Assay

The effects of the medium with different glucose concentrations on the viability of PDLFs were assessed in triplicate wells in three independent experiments using the Alamar blue assay. First, cells were seeded in 96-well plates (Thermo Fisher Scientific) at 5 × 10^4^ cells per well in 200 µL of complete culture medium and grown overnight in a cell culture incubator (37 °C, 100% humidity, 95% air, and 5% CO_2_) to allow adherence to the plate surface. On the next day, the non-adherent cells were removed by aspiration. Next, adherent cells were subjected to culture media with three glucose concentrations (5.5, 25, and 50 mM) with or without 1 µg/mL of LPS [19,24]. After 24 and 48 h, cells were incubated for 4 h with 20 µL of Alamar blue dye (Thermo Fisher Scientific) in a standard cell culture incubator. The fluorescence was measured at 590 nm using BioTek^®^ Synergy™ HT Microplate Reader (BioTek^®^, Winooski, VT, USA).

### 2.5. Lactic Acid Dehydrogenase (LDH) Cytotoxicity Assay

The cytotoxicity effects of different glucose concentrations were measured by colorimetric method using an LDH assay kit (Elabscience^®^ Biotechnology). PDLF cells were seeded in 96-well plates (Thermo Fisher Scientific) at 5 × 10^4^ cells per well in 200 µL of complete culture medium and incubated at (37 °C, 100% humidity, 95% air, and 5% CO_2_) upon confluency. Next, culture media containing three different glucose concentrations (5.5, 25, and 50 mM) with or without 1 µg/mL of LPS were added to each appropriate experimental well. After 24 and 48 h, two independent experiments in triplicate wells were carried out following the manufacturer’s protocol. The optical densities of each well were assessed at 450 nm using BioTek^®^ Synergy™ HT Microplate Reader (BioTek^®^).

### 2.6. Scratch Migration Assay

The wound closure ability of PDLFs in different glucose concentrations was measured using the scratch migration assay. PDLFs cells were plated on 12-well culture plates at a density of 5 × 10^4^ cells/well, incubated in a complete culture medium, and grown overnight at 37 °C, 100% humidity, 95% air, and 5% CO_2_ to achieve confluency. A sterile ruler was used to reference the center, then a scratch was manually produced across the cell monolayer using a sterile p200 pipette tip (Thermo Fisher Scientific). Cells were then washed with PBS to remove cellular debris and exposed to each glucose concentration with/without 1 µg/mL of LPS. After scratching, the wound closure was observed at baseline, 6, 24, and 48 h under a phase-contrast microscope (Carl Zeiss Axiovert 40C Imaging Microscope, Göttingen, Germany) until the wound healed in all the groups, and digital photographs were captured by matching reference points. The percent wound closure was calculated using ImageJ software (National Institutes of Health, Bethesda, MD, USA) version 1.50i. Data are representative of 3 replicas for each experimental condition, where 3 images were captured per condition.

### 2.7. Quantification of Inflammatory Cytokines Levels by Real-Time Quantitative Polymerase Chain Reaction (RT-qPCR)

PDLFs were seeded in 6-well plates at a density of 1 × 10^6^ cells per well in a standard cell culture incubator (37 °C, 100% humidity, 95% air, and 5% CO_2_). After 24 h, non-adherent cells were removed, and 2 mL of culture media containing three different glucose concentrations (5.5, 25, and 50 mM) were added. Cells were then stimulated with or without 1 µg/mL of LPS. The experiments were performed three times on separate days in triplicate wells for each group to validate the data and ensure reproducibility.

After 6 and 24 h, total RNA was extracted using the HiGene^TM^ Total RNA prep kit (BioFACT, Daejeon, Republic of Korea) according to the manufacturer’s instructions. Briefly, cell pellets were lysed with β-mercaptoethanol and proteinase K. After that, they were vortexed, incubated (56° C, 10 min), then centrifuged at 14,000× *g* for 3 min, and ethanol (100%) was then added. The mixture was vortexed for 30 s. The RNA was eluted with 50 μL of RNase-free water. Next, the RNA concentration and quality were evaluated using the Eppendorf Biospectrophotometer Plus (Eppendorf GA, Hamburg, Germany). Finally, the reverse transcription of samples was performed to obtain the complementary DNA (cDNA) synthesized from 500 ng of RNA using a cDNA synthesis kit (Solis Biodyne, Tartu, Estonia).

The expression of IL-6, IL-10, IL-23 (p19/p40), and TLR-4 in PDLFs was quantified using RT-qPCR with 5× HOT FIREPol^®^ EvaGreen qPCR Supermix (Solis Biodyne) on an ABI 7500 real-time PCR system (Applied Biosystems, Foster City, CA, USA). Reactions were carried out in a volume of 20 µL containing 1 µg of cDNA and primers under the following thermal conditions: 95 °C for 12 min followed by 40 cycles of 95 °C for 15 s, 65 °C for 30 s, and 72 °C for 30 s. The threshold cycle (Ct) value for each target gene was normalized to the Ct value for the reference gene glyceraldehyde-3-phosphate dehydrogenase (GAPDH), and relative expression was expressed as fold change using the formula 2^−ΔΔCt^ as previously described by Alsalleeh et al. [32]. The forward and reverse primers (Macrogen, MD, USA) used in the experiment are shown in Table 1.

### 2.8. Quantification of Interleukin-6 and Interleukin-10 by Enzyme-Linked Immunosorbent Assay (ELISA)

According to the manufacturer’s instructions, the proteins secreted by PDLFs of IL-6 and IL-10 in cell culture supernatants after 48 h of exposure to each glucose concentration with/without 1 µg/mL of LPS were quantified with ELISA kits (Elabscience^®^ Biotechnology). Briefly, diluted 100 µL samples and standards were added onto a 96-well plate and incubated for 2 h at 37 °C. Upon aspiration, 100 µL of biotinylated detection Ab working solution was added to each well and incubated for 2 h at 37 °C. The solutions were discarded and washed with wash buffer, and 100 µL of HRP conjugate working solution was added to the wells and incubated for 1 h at 37 °C. The wells were decanted and rewashed with wash buffer. After washing, 90 µL of substrate reagent was added and incubated for 30 min at 37 °C. Then, 50 µL of stop solution was added to each well, and the optical density at 450 nm was measured using BioTek^®^ Synergy™ HT Microplate Reader (BioTek^®^). Two independent experiments were performed in triplicate wells for each group to validate the data and ensure reproducibility.

### 2.9. Statistical Analysis

The results are presented as mean ± standard error of the mean of three independent experiments. Using SPSS software (SPSS Inc., Chicago, IL, USA) version 23, the data were evaluated by univariate analysis of variance (ANOVA) followed by Tukey’s post-hoc test for multiple comparisons. Values were considered statistically significant when *p* < 0.05. Graphs were created with GraphPad Prism 9 software (GraphPad Software, San Diego, CA, USA).

## 3. Results

### 3.1. PDLFs Express Vimentin and Lack Keratin

**Figure 1:**
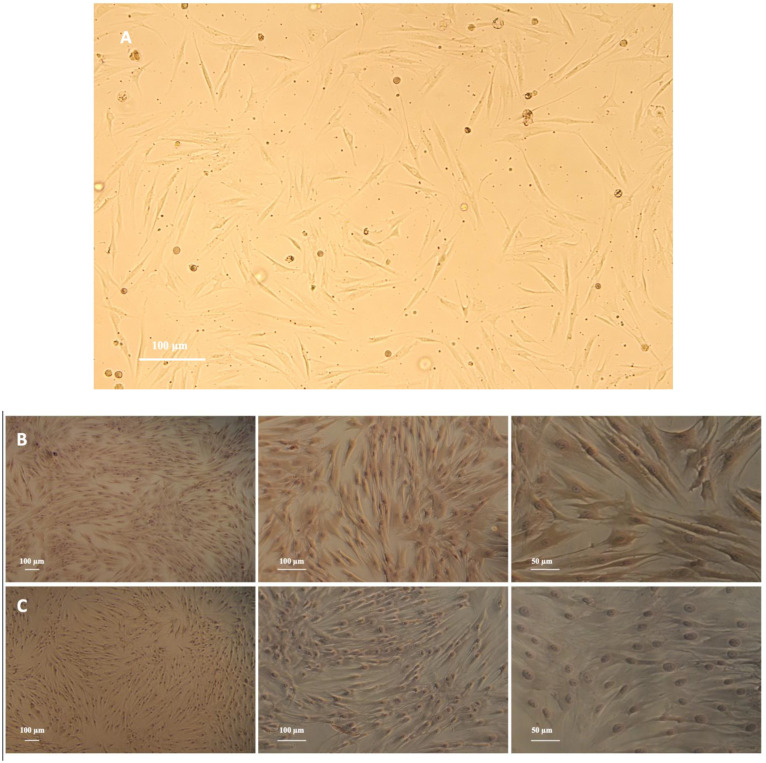
(**A**) Primary culture of periodontal ligament fibroblasts (PDLFs) (magnification, ×10), scale bar: 100 μm. PDLFs are spindle in shape and arranged in a radial pattern. (**B**) Identification of periodontal ligament fibroblasts (PDLFs) using immunohistochemistry (IHC): detection of anti-vimentin expression in PDLFs (magnification, ×5, ×10, ×20), scale bar: 100 and 50 μm. The PDLFs’ cytoplasm is stained brown. (**C**) Identification of periodontal ligament fibroblasts (PDLFs) using immunohistochemistry (IHC): detection of anti-keratin expression in PDLFs (magnification, ×5, ×10, ×20), scale bar: 100 and 50 μm. Positive staining was not observed.

PDLFs were spindle shaped and arranged in a radial pattern (Figure 1A). Immunohistochemical characterization revealed that PDLFs had positive anti-vimentin staining and negative anti-keratin staining (Figure 1B,C).

### 3.2. The Proliferation of PDLFs Was Affected by Different Glucose Concentrations

**Figure 2:**
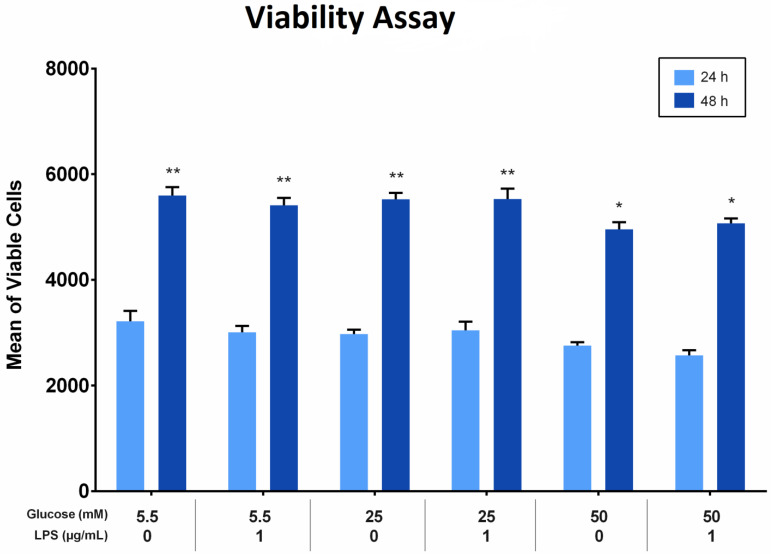
The viability of periodontal ligament fibroblast (PDLFs) using Alamar blue assay at 24 and 48 h. The cells were challenged with glucose concentrations with/without 1 µg/mL LPS. Asterisks (**) indicate *p* < 0.05 compared with all other groups with/without LPS at 24 and 48 h. Asterisk (*) indicates *p* < 0.05 compared with 5.5 mM, 25 mM, and 50 mM glucose with/without LPS at 24 h. (LPS; lipopolysaccharide).

The effect of different glucose concentrations with and without LPS stimulation on the proliferation of PDLFs was analyzed using the Alamar blue assay. All groups showed more viable cells at 48 h than at 24 h (*p* < 0.05). At 24 and 48 h, the viability of cells treated with 50 mM glucose was significantly fewer compared to other glucose concentrations, regardless of LPS stimulation (*p* < 0.05). Furthermore, the viable cells treated with 25 mM glucose were comparable to those treated with 5.5 mM glucose at the same time, at 24 and 48 h. The difference was insignificant (*p* > 0.05). Factorial ANOVA showed that LPS did not significantly influence PDLF proliferation in all groups. Moreover, there was no interaction effect of different glucose levels and LPS on cell proliferation (*p* > 0.05; Figure 2).

### 3.3. High-Glucose Concentrations Amplified the Cytotoxicity of PDLFs

**Figure 3:**
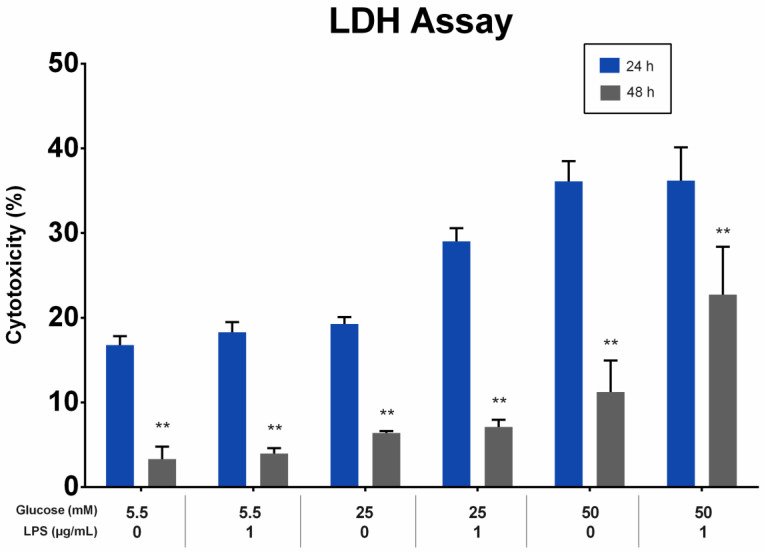
The cytotoxicity effect on periodontal ligament fibroblast (PDLFs) using LDH assay at 24 and 48 h. The cells were challenged with glucose concentrations with/without 1 µg/mL LPS. Asterisks (**) indicate *p* < 0.01 compared with the same glucose concentration at 24 h. (LDH; Lactic Acid Dehydrogenase, LPS; lipopolysaccharide).

The effect of different glucose concentrations with and without LPS stimulation on the cytotoxicity of PDLFs was analyzed using the LDH assay. LDH levels in the supernatant were directly proportional to the glucose concentration at 24 and 48 h. All groups showed a greater reduction in LDH levels at 48 h than at 24 h (*p* < 0.01). At 24 and 48 h, the LDH levels of PDLFs treated with 50 mM glucose were higher than other glucose concentrations, regardless of LPS stimulation (*p* < 0.05). The LPS stimulation of the 50 mM glucose-treated PDLFs resulted in a significant increase in LDH level compared to no LPS-stimulated cells at 48 h (*p* < 0.05; Figure 3).

### 3.4. High-Glucose Concentrations Have Adverse Effects on PDLFs Wound Closure

**Figure 4:**
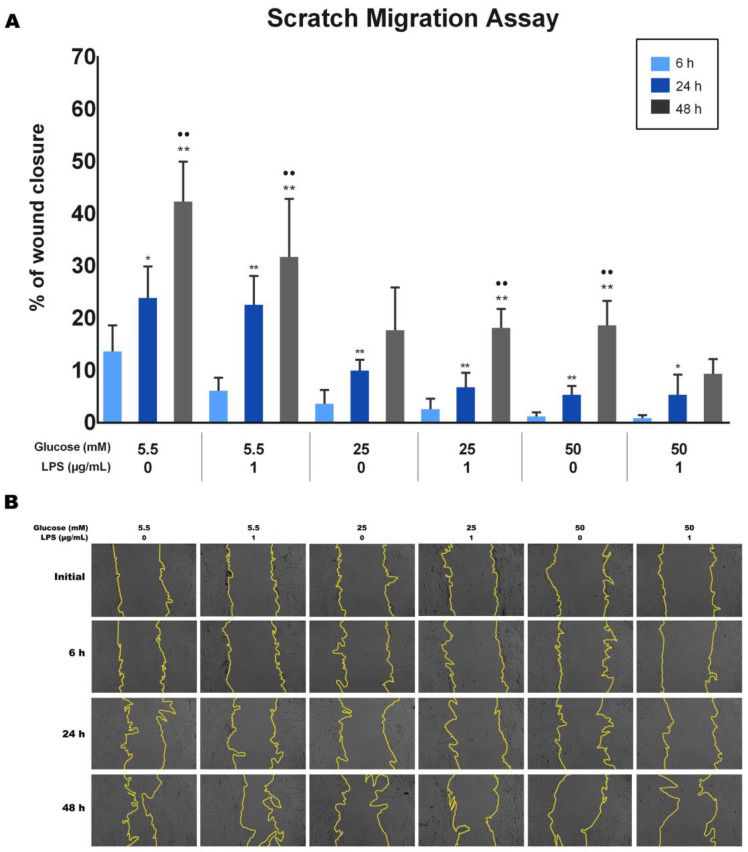
The migration ability of periodontal ligament fibroblasts (PDLFs) using scratch migration assay at 6, 24, and 48 h. The cells were challenged with glucose concentrations with/without 1 µg/mL LPS. The wound closure percentage is shown in (**A**), and representative images are shown in (**B**). Dots (**..**) indicate *p* < 0.05 compared to the same group at 24 h. Asterisks (**) indicate *p* < 0.05, and compared to the same group at 6 h. Asterisk (*) indicates *p* < 0.01 compared to the same group at 6 h. Scale bar: 50 μm. (LPS; lipopolysaccharide).

The effect of different glucose concentrations with and without LPS stimulation on the wound closure ability of PDLFs was analyzed using the scratch wound assay. Wound closure was quantified, and the results are shown in Figure 4A. Results indicate that the wound healing percentages significantly increased with time for each group (*p* < 0.05). The 5.5 mM glucose concentration with/without LPS appeared to have the highest percentage of wound closure among all groups at 6, 24, and 48 h (*p* < 0.05). At the same time, 25 mM and 50 mM glucose concentrations significantly decreased the migration ability of PDLFs compared to 5.5 mM, regardless of LPS stimulation (*p* < 0.05). Furthermore, cells treated with 50 mM glucose with LPS at 48 h exhibited the least migration ability among all groups (*p* < 0.05). Representative images are presented in Figure 4B.

### 3.5. LPS Induces the Expression of Interleukin-6, Interleukin-23, and Toll-like Receptor-4 but Not Interleukin-10

**Figure 5:**
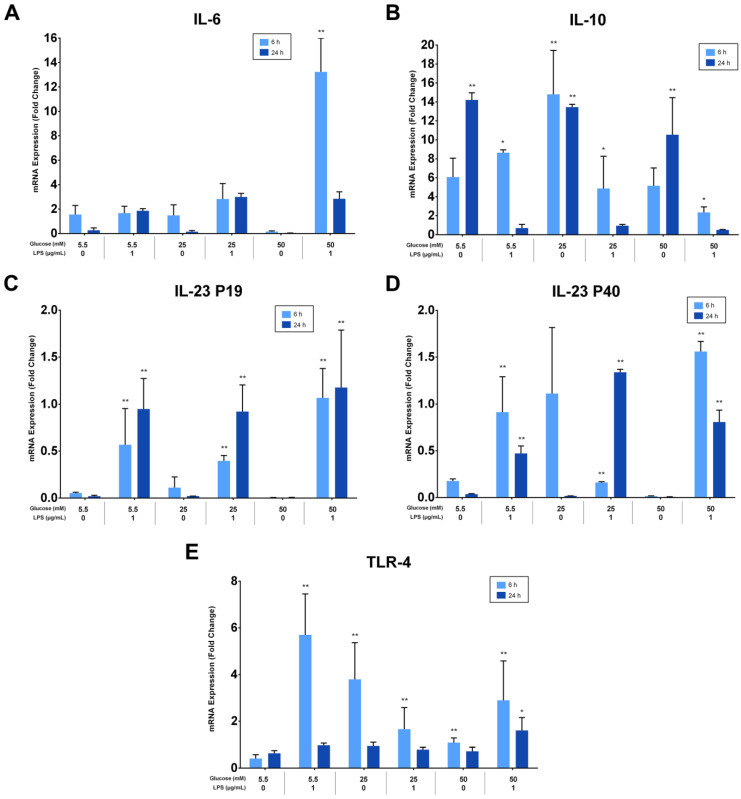

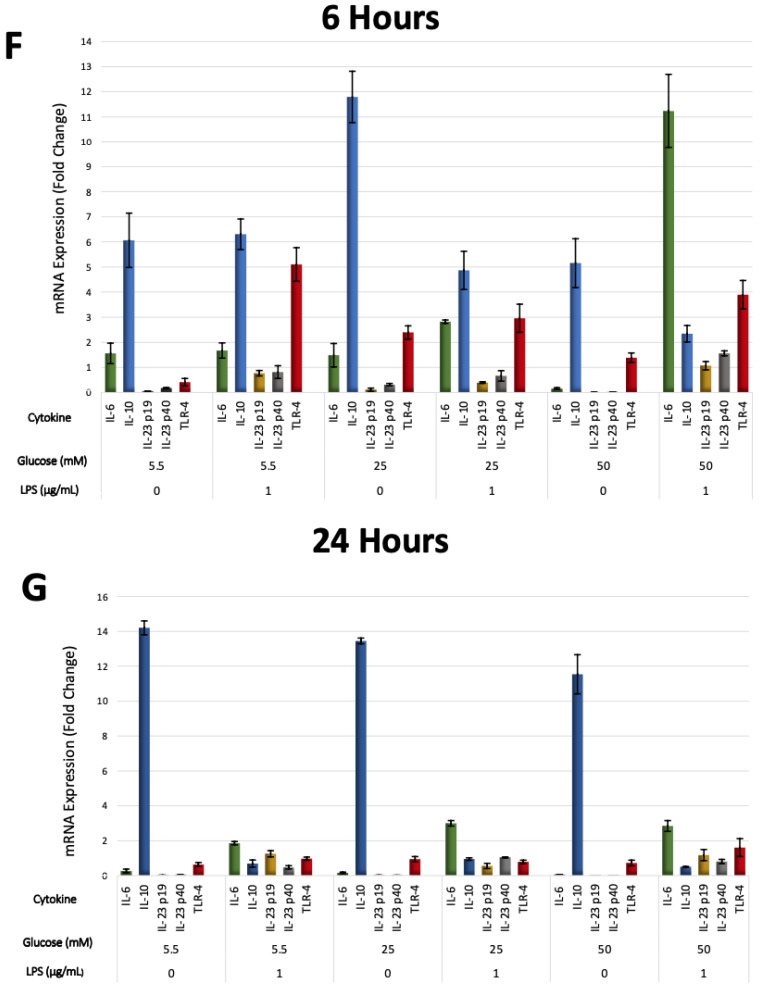
(**A**) IL-6 mRNA expression (fold change) at 6 and 24 h. IL-6 was significantly up-regulated at 6 h in 50 mM glucose with LPS-stimulated cells (*p* < 0.05). Asterisks (**) indicate *p* < 0.01 compared with all other IL-6 expressions at different times and glucose concentrations. (IL-6; Interleukin-6, mRNA; messenger RNA, LPS; lipopolysaccharide). (**B**) IL-10 mRNA expression (fold change) at 6 and 24 h. IL-10 was significantly up-regulated in LPS-free cells (*p* < 0.05). Asterisks (**) indicate *p* < 0.05 compared with all other IL-10 expressions. Asterisk (*) indicates *p* < 0.05 compared with IL-10 expressions in LPS-stimulated groups at 24 h. (IL-10; Interleukin-10, mRNA; messenger RNA, LPS; lipopolysaccharide). (**C**,**D**) IL-23 p19/p40 expression (fold change) at 6 and 24 h. IL-23 p19/p40 was significantly up-regulated in LPS-challenged cells (*p* < 0.05). Asterisks (**) indicate *p* < 0.05 compared with all other IL-23 p19/p40 expressions without LPS stimulation. (IL-23 p19; Interleukin-23 p19, IL-23 p40; Interleukin-23 p40, mRNA; messenger RNA, LPS; lipopolysaccharide). (**E**) TLR-4 expression (fold change) at 6 and 24 h. TLR-4 was significantly up-regulated in LPS-stimulated cells and in 25 and 50 mM glucose-treated cells at 6 h (*p* < 0.05). Asterisks (**) indicate *p* < 0.05 compared with TLR-4 expressions at 24 h in the same group. Asterisk (*) indicates *p* < 0.05 compared with all other TLR-4 expressions at 24 h. (TLR-4; Toll-like receptor-4, mRNA; messenger RNA, LPS; lipopolysaccharide). (**F**) Six-hour mRNA expressions (fold change) of IL-6, IL-10, IL-23 (p19/p40), and TLR-4. (IL-6; Interleukin-6, IL-10; Interleukin-10, IL-23 p19; Interleukin-23 p19, IL-23 p40; Interleukin-23 p40TLR-4; Toll-like receptor-4, mRNA; messenger RNA, LPS; lipopolysaccharide). (**G**) Twenty-four-hour mRNA expressions (fold change) of IL-6, IL-10, IL-23 (p19/p40), and TLR-4. (IL-6; Interleukin-6, IL-10; Interleukin-10, IL-23 p19; Interleukin-23 p19, IL-23 p40; Interleukin-23 p40TLR-4; Toll-like receptor-4, mRNA; messenger RNA, LPS; lipopolysaccharide).

The effects of different glucose concentrations on the expressions of PDLFs of IL-6, IL-10, IL-23 (p19/p40), and TLR-4 after LPS stimulation were investigated using RT-qPCR, analyzed at 6 and 24 h, and normalized to GAPDH. IL-6 was significantly up-regulated by LPS-stimulated cells in a 50 mM glucose medium at 6 h (*p* < 0.05). Lower glucose concentrations had low, yet detectable IL-6 expressions at 6 and 24 h (*p* > 0.05; Figure 5A)**.** Moreover, IL-10 was constitutively expressed under different glucose concentrations and down-regulated in LPS-stimulated cells (*p* < 0.05). The expression of IL-10 in LPS-free cells was significantly greater at 24 h than 6 h (*p* < 0.05), except for the 25 mM glucose medium, where it was comparable (Figure 5B). Although different glucose concentrations did not significantly influence the expression of IL-23 subunits (*p* > 0.05), it was up-regulated after LPS stimulation at 6 and 24 h (*p* < 0.05). The expression of IL-23 p19 was amplified insignificantly after LPS stimulation in 50 mM glucose concentration at 6 and 24 h (*p* > 0.05). Likewise, the IL-23 p40 was highly expressed after LPS stimulation in 50 mM glucose concentration at 6 h (*p* < 0.05; Figure 5C,D). TLR-4 was up-regulated after LPS stimulation regardless of glucose concentrations at 6 h (*p* < 0.05). However, cells in the absence of LPS own low but detectable levels of TLR-4 at 6 and 24 h (*p* > 0.05; Figure 5E). The expressions of PDLFs of IL-6, IL-10, IL-23 (p19/p40), and TLR-4 at 6 h are represented in (Figure 5F) and 24 h in (Figure 5G).

### 3.6. LPS and High-Glucose Concentrations Induce Interleukin-6 Secretion but Not Interleukin-10

**Figure 6:**
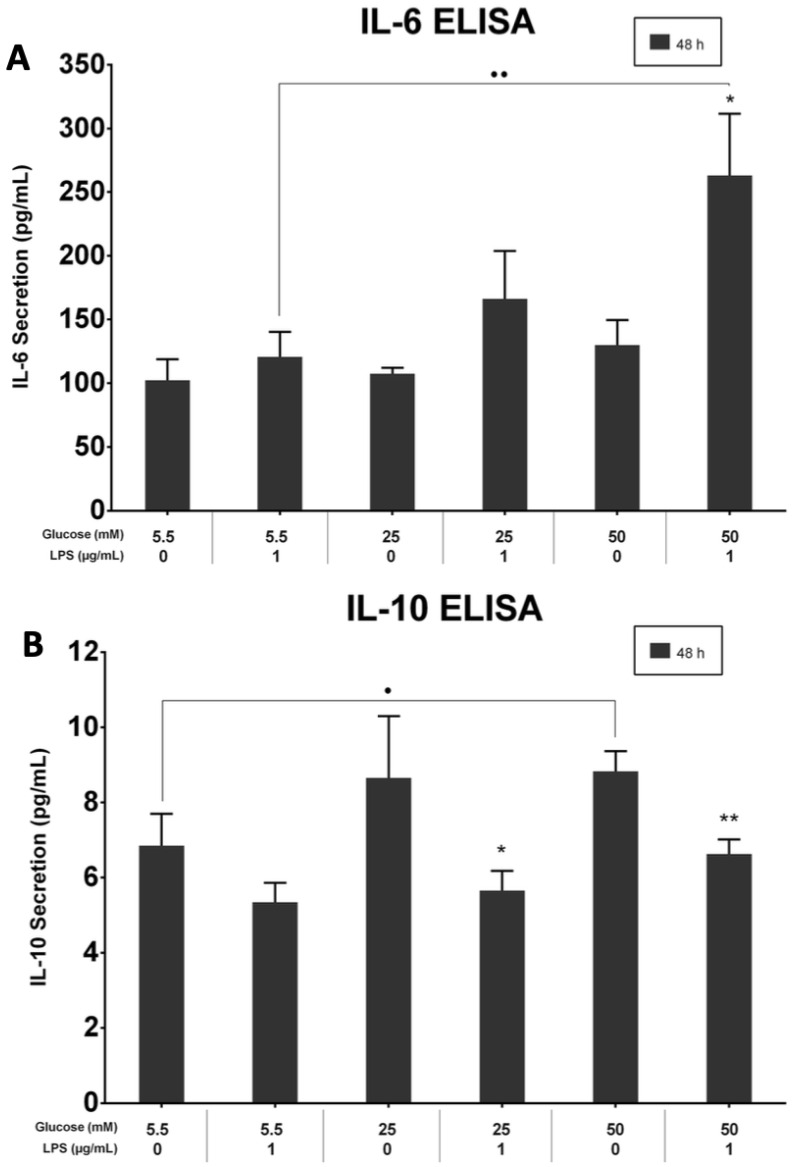
(**A**) IL-6 secretion at 48 h. IL-6 was significantly up-regulated in LPS-stimulated and 50 mM glucose-treated cells at 48 h (*p* < 0.05). Asterisk (*) indicates *p* < 0.05 compared with IL-6 secretion in the 50 mM glucose concentration without LPS stimulation. Dots (**..**) indicate *p* < 0.01 compared to different glucose concentrations with LPS stimulation. (IL-6; Interleukin-6, ELISA; Enzyme-linked immunosorbent assay, LPS; lipopolysaccharide). (**B**) IL-10 secretion at 48 h. IL-10 was significantly up-regulated in LPS-free cells at 48 h (*p* < 0.05). Asterisks (**) indicate *p* < 0.01 compared with IL-10 secretion in 50 mM glucose without LPS stimulation. Asterisk (*) indicates *p* < 0.05 compared with IL-10 secretion in 25 mM glucose without LPS stimulation. Dot (**.**) indicates *p* < 0.05 compared to different glucose concentrations with LPS stimulation. (IL-10; Interleukin-10, ELISA; Enzyme-linked immunosorbent assay, LPS; lipopolysaccharide).

The effects of different glucose concentrations on IL-6 and IL-10 supernatant levels in PDLFs were quantified with ELISA kits. LPS-stimulated cells in the 50 mM glucose concentration secreted the highest level of IL-6 at 48 h (*p* > 0.05). The IL-6 protein level in 5.5 mM and 25 mM glucose concentrations was significantly up-regulated by LPS stimulation than those without LPS at 48 h (*p* > 0.05; Figure 6A). Moreover, IL-10 was constitutively secreted under different glucose concentrations, and the secretions down-regulated in the LPS-stimulated cells (*p* < 0.05; Figure 6B).

The effects of different glucose concentrations on IL-6 and IL-10 supernatant levels in PDLFs were quantified with ELISA kits. The LPS-stimulated cells in 50 mM secreted the highest level of IL-6 at 48 h (*p* > 0.05). The IL-6 protein level in 5.5 mM and 25 mM was significantly up-regulated by LPS stimulation than those without LPS at 48 h (*p* > 0.05; Figure 6A). Moreover, IL-10 was constitutively secreted under different glucose concentrations, and the secretions were down-regulated in the LPS-stimulated cells (*p* < 0.05; Figure 6B).

## 4. Discussion

DM is a risk factor associated with a greater prevalence and severity of periodontitis [35]. Both animal and clinical studies demonstrated a well-recognized bidirectional relationship between DM and periodontal disease [36]. On a cellular bases, evidence has shown that hyperglycemic conditions influence the biological and inflammatory response of PDLFs [19,22,23,24]. Therefore, to advance the current understanding of the pathogenic mechanism of periodontitis in diabetic patients, the present study aimed to compare the effect of 5.5 mM glucose concentrations with high- and severely high-glucose concentrations, 25 mM and 50 mM, respectively, on PDLFs in the presence of the bacterial stimulus, the LPS.

The results herein revealed that the 50 mM glucose concentration in the LPS-free medium significantly suppressed the proliferation capacity of PDLFs compared with cell proliferation under 5.5 and 25 mM glucose concentrations. However, LPS stimulation at 1 µg/mL did not affect cell viability under all glucose concentrations. Studies have shown that 1 µg/mL of LPS is the optimum concentration to induce inflammatory responses [18,31,37]. This finding agrees with a previous study with a similar experimental design. Notably, these authors found that LPS at 1 µg/mL did not influence cell viability. In contrast, LPS at 10 µg/mL induced significant apoptosis in PDLFs and reduced their viability, where this effect was amplified in a 25 mM glucose medium [19]. The present study found that the proliferation ability of cells treated with 25 mM glucose was not significantly affected at 24 and 48 h. A previous study reported a similar outcome [24]. However, other studies reported that a prolonged incubation period (4–14 days) at high-glucose concentrations (25 and 30 mM) significantly suppressed cell proliferation and increased cell apoptosis compared to treatment with 5.5 mM glucose [23,38,39]. It appears that the viability and proliferation ability of PDLFs under high-glucose conditions are significantly affected in a time- and concentration-dependent manner.

The apoptosis and cytotoxicity effect of hyperglycemia on PDLFs has been an area of interest. PDLFs can induce apoptosis through the caspase-3 signaling pathway [40,41]. As the previous study presented, more apoptotic cells were observed due to 25 mM glucose combined with 10 mg/mL of LPS at 24 h or 48 h [19]. Moreover, the percentage of apoptosis-positive cells increased significantly with time in high-glucose concentrations [42]. LDH levels in supernatant are a hallmark of cytotoxicity. The present study has shown that increased LDH is proportional to glucose concentration. Additionally, a 50 mM glucose concentration significantly increased LDH levels, and the LPS stimulation amplified its effect. Our results align with previous investigations that showed that human gingival fibroblasts treated with 50 mM and 75 mM glucose for 72 h resulted in greater cellular damage. In comparison, 25 mM glucose did not significantly change LDH levels [43].

The results of the present study show that 25 mM and 50 mM glucose concentrations significantly impair the migration ability of PDLFs, regardless of the 1 µg/mL LPS stimulation. Nevertheless, 1 µg/mL of LPS amplified the 50 mM glucose effects and exhibited the least migration ability at 48 h. A previous study reported that human gingival fibroblasts treated with 50 mM and 75 mM glucose for 72 h significantly showed slower migration at 48 h, resulting in prolonged wound closure [43].

It is well known that periodontitis aggravates the production of systemic inflammatory mediators. The presence of these mediators in the systemic circulation under a hyperglycemic state amplifies inflammation and accelerates apoptosis and oxidative stress associated with diabetes [11,30]. The current study evaluated selected inflammatory mediators after subjecting the PDLFs to different glucose concentrations in the presence or absence of LPS.

IL-6 is a multifunctional cytokine that acts as a pro- and anti-inflammatory [44]. PDLFs significantly up-regulated IL-6 expression in response to LPS stimulation in the 50 mM glucose medium at 6 h. Previous findings demonstrated that, at 6 h, cells treated with 45 mM glucose showed significantly higher IL-6 expression than those treated with 25 mM glucose [20]. Another study revealed that IL-6 levels were increased considerably, mainly after 72 h in cells treated with 12 and 24 mM glucose [45]. However, in these studies, PDLFs were not stimulated with LPS. These data suggest that the presence of excessive glucose induces IL-6 expression, especially in the presence of microbial stimulation.

IL-10, a crucial anti-inflammatory cytokine, was constitutively expressed by PDLFs under different glucose concentrations, but it was inhibited in response to LPS stimulation. The results here agree with a previous study that showed 45 mM glucose up-regulated the IL-10 expression in bone marrow-derived macrophages, whereas 1 µg/mL of LPS stimulation down-regulated IL-10 expression [46]. The effect of LPS on IL-10 expression needs further investigation, which is beyond the objective of the present study.

IL-23 is an essential pro-inflammatory cytokine required for the survival and expansion of the T-helper 17 cells and the production of IL-17 [47]. Moreover, IL-23 is well known to play a significant role in diabetes and periodontitis [48,49]. The results demonstrated that IL-23 subunits were expressed after LPS stimulation, especially under severe high-glucose conditions. Therefore, we can infer from the present study that hyperglycemic conditions might favor the development of an IL-17 phenotype of T-cells.

The present study highlighted that PDLFs respond differently to various glucose concentrations. Indeed, hyperglycemia plays an integral role in the pathogenesis and progression of periodontal disease in diabetic patients. Given the limitations of this in vitro study, such as the use of a single concentration of LPS, and the constant high-glucose condition for a relatively short time, further in vitro studies with different cell lines and stimuli and in vivo investigations involving periodontitis under diabetic conditions are required to verify and generalize the findings.

## 5. Conclusions

Severely high- glucose (50 mM) concentrations decreased the proliferation and migration activity of PDLFs, increased cellular cytotoxicity, and induced the expression of IL-6 and IL-23 inflammatory mediators associated with periodontitis.

## Figures and Tables

**Table 1:** Primer sequences.

Target	Forward Primer Sequence (5′-3′)	Reverse Primer Sequence (5′-3′)
IL-6	5′-AGGAGACTTGCCTGGTGAAA-3′	5′-CAGGGGTGGTTATTGCATCT-3′
IL-10	5′-TGGTGAAACCCCGTCTCTAC-3′	5′-CTGGAGTACAGGGGCATGAT-3′
IL-23 P19	5′-GTTCCCCATATCCAGTGTGG-3′	5′-TTTTGAAGCGGAGAAGGAGA-3′
IL-23 P40	5′-ACGGACAAGACCTCAGCCAC-3′	5′-GGGCCCGCACGCTAA-3′
TLR-4	5′-ACAACCTCCCCTTCTCAACC-3′	5′-TGAGATGTCCAATGGGGAAG-3′
GAPDH	5′-CAGCCTCCCGCTTCGCTCTC-3′	5′-CCAGGCGCCCAATACGACCA-3′

Interleukin-6, IL-6; Interleukin-10, IL-10; Interleukin-23 P19, IL-23 P19; Interleukin-23 P40, IL-23 P40; Toll-like receptor, TLR-4; Glyceraldehyde-3-phosphate dehydrogenase, GAPDH. Data are according to [32,33,34].

## Data Availability

Data are contained within the article.
